# Colistin-resistant *Escherichia coli* harboring *mcr-1* in slaty-backed gull breeding in Northern Japan

**DOI:** 10.1128/spectrum.00703-24

**Published:** 2024-10-31

**Authors:** Takehiko Kenzaka, Yuto Tsuruta, Yuuka Nakamura, Shouta Ueda

**Affiliations:** 1 Faculty of Science and Engineering, Setsunan University, Neyagawa, Osaka, Japan; 2 Faculty of Pharmacy, Osaka Ohtani University, Tondabayashi, Osaka, Japan; University of Mississippi, University, Mississippi, USA

**Keywords:** colistin resistance, *Escherichia coli*, avian, bird, *Larus schistisagus*, *mcr-1*, gull

## LETTER

Colistin serves as a crucial antibiotic, often employed as a final recourse against severe infections induced by multidrug-resistant gram-negative bacteria. Nonetheless, the rising incidence of colistin-resistant bacteria carrying the mobile colistin resistance (mcr) gene presents a significant challenge to effective treatment (1). Surveillance of colistin resistance and the mobilized resistance genes has been conducted for Enterobacteriaceae isolated from animals such as poultry and pig, foods, and humans (2–6). Additionally, *mcr-1* has been found in fecal bacteria from European herring gulls (*Larus argentatus*), yellow-legged gulls (*Larus michahellis*), and kelp gulls (*Larus dominicanus*) in Europe and South America. Gulls are suspected to serve as reservoir and bridge hosts for disseminating *mcr-1* among poultry, livestock, and humans (7–10).

Slaty-backed gulls (*Larus schistisagus*) breed from spring to summer in Hokkaido, the northern part of Japan, and migrate to the middle part of Japan, southern areas of East Asia, and Southeast Asia (11). Migratory birds may play an important role as vectors in spreading antibiotic-resistant bacteria across geographically distant areas (12). In this study, we found that most of the *L. schistisagus* breeding in the northern part of Japan carried commensal colistin-resistant *Escherichia coli* harboring *mcr-1*. As far as we are aware, this is the first report of high colistin-resistant *E. coli* contamination in *L. schistisagus* breeding around East Asia.

A total of 238 fecal specimens were collected from three breeding areas on Teuri Island, Wakkanai, and Akkeshi in Hokkaido, Japan, between 2018 and 2022. Only fresh droppings were collected under conditions where no other birds were present. The samples were plated onto CHROMagar COL-APSE (CHROMagar, Paris, France) for the isolation of colistin-resistant *E. coli*. Representative *E. coli* colonies were singled out and subjected to further characterization. Antibiotic susceptibilities to amikacin, azithromycin, cefotaxime, ceftazidime, ciprofloxacin, colistin, gentamicin, imipenem, meropenem, and tetracycline were determined using the Etest (bioMérieux Japan Ltd., Tokyo, Japan) in accordance with the manufacturer’s instructions. The *mcr* genotype in the isolates was examined by multiplex PCR (13, 14), and the presence of *mcr-1* in fecal samples was confirmed using real-time PCR (15). Nearly full-length 16S rRNA gene and *mcr-1* were amplified by PCR using primers 16S_8f (5′-AGAGTTTGATCMTGGCTCAG-3′) and 16S_1492r (5′-TACGGYTACCTTGTTACGACTT-3′), and mcr1-up1-2 (5′-TGCCGTAATTATCCCACCG-3′) and mcr1-dw1-2 (5′-ACCAATCAGCGACCATC-3′), supplemented with the universal tails 5′-TTTCTGTTGGTGCTGATATTGC-3′ (forward), 5′-ACTTGCCTGTCGCTCTATCTTC-3′ (reverse). The PCR products were processed using the SQK-LSK109 kit with barcoding via the EXP-PBC096 kit (Oxford Nanopore Technologies, Oxford, UK), and the sequencing was performed with the MinION Mk1C (Oxford Nanopore Technologies). The trimmed reads were assembled with the appropriate references using CodonCode Aligner version 5.1.5 (CodonCode, Inc., Centerville, MA, USA).

As shown in Table 1, fecal samples from *L. schistisagus* at all three sites exhibited the presence of *mcr-1*, with 36% of total fecal samples testing positive. The difference in positive rates among the sites might be due to differences in diet organisms, which may contain colistin-resistant *E. coli*. Overall, COL-APSE-positive samples were more prevalent than *mcr-1*-positive samples. The discrepancy between the occurrence of the genotype (*mcr-1*) and the phenotype (COL-APSE) may be attributed to other genotypes or mechanisms. Although MCR family proteins catalyze the modification of lipid A, colistin resistance can also result from the loss of lipid A, the activity of drug efflux pumps, or chromosomal point mutations (16). The median minimum inhibitory concentration (MIC) of colistin for the colistin-resistant *E. coli* isolates was 12 mg L^−1^ (Fig. S1). In the multiplex PCR analysis of *mcr-1* to *-9* in the isolates, *mcr-1* was detected at a rate of 81.1%, while *mcr-2* to *-9* were not detected. The response of colistin-resistant *E. coli* isolates to antibiotics indicated that 8.8% exhibited resistance to colistin, azithromycin (median MIC of 24 mg L^−1^), and tetracycline (median MIC of 256 mg L^−1^) at the same time, while both colistin and tetracycline resistance was detected in 75.5% of cases. The *L. schistisagus* travels an average of 25 km daily within its geographic range for foraging, with some individuals covering up to 100 km (17). In the feeding area studied here, there are sewage treatment plants and landfills, which might be sources of *mcr-1*. The longest migration distance recorded exceeds 4,000 km. These results suggest that migratory gulls play a role in the dissemination of colistin-resistant *E. coli* in natural environments. Therefore, there is an urgent need to implement the One Health approach to prevent the spread of antibiotic-resistant bacteria.

**TABLE 1:** Detection of colistin-resistant *E. coli* harboring *mcr-1* in fecal specimens of slaty-backed gull (*Larus schistisagus*)

*E. coli* Phenotype^*a*^ or genotype^*b*^	No. of positive specimens/no. of specimens tested			
	All sites (*n* = 317)	Site TE (*n* = 238)	Site WA (*n* = 30)	Site AK (*n* = 49)
COL-APSE^*a*^	58.1%	50.6%	80.6%	70.6%
*mcr-1^b^*	36.6%	35.7%	26.7%	46.9%

^*a*^ COL-APSE.

^*b*^ *mcr-1.*

## Data Availability

The 16S rRNA gene and *mcr-1* sequences of isolates have been deposited in the DDBJ/EMBL/GenBank database under accession nos. PQ097308-PQ097500 and PQ141725-PQ141848, respectively.

## SUPPLEMENTAL MATERIAL

Figure S1 spectrum.00703-24-s0001.pdf Distribution of colistin MICs in isolates from Slaty-backed Gulls.

ASM does not own the copyrights to Supplemental Material that may be linked to, or accessed through, an article. The authors have granted ASM a non-exclusive, world-wide license to publish the Supplemental Material files. Please contact the corresponding author directly for reuse.

## References

[1] Paterson DL, Harris PNA. 2016. Colistin resistance: a major breach in our last line of defence. Lancet Infect Dis 16:132–133. doi:10.1016/S1473-3099(15)00463-6 26603171

[2] Liu YY, Wang Y, Walsh TR, Yi LX, Zhang R, Spencer J, Doi Y, Tian G, Dong B, Huang X, Yu LF, Gu D, Ren H, Chen X, Lv L, He D, Zhou H, Liang Z, Liu JH, Shen J. 2016. Emergence of plasmid-mediated colistin resistance mechanism MCR-1 in animals and human beings in China: a microbiological and molecular biological study. Lancet Infect Dis 16:161–168. doi:10.1016/S1473-3099(15)00424-7 26603172

[3] Makita K, Fujimoto Y, Sugahara N, Miyama T, Usui M, Asai T, Kawanishi M, Ozawa M, Tamura Y. 2020. Quantitative release assessment of mcr-mediated colistin-resistant Escherichia coli from Japanese Pigs. Food Saf (Tokyo) 8:13–33. doi:10.14252/foodsafetyfscj.D-20-00004 32626634 PMC7329916

[4] Kawamoto Y, Kaku N, Akamatsu N, Sakamoto K, Kosai K, Morinaga Y, Ohmagari N, Izumikawa K, Yamamoto Y, Mikamo H, Kaku M, Oishi K, Yanagihara K. 2022. The surveillance of colistin resistance and mobilized colistin resistance genes in multidrug-resistant Enterobacteriaceae isolated in Japan. Int J Antimicrob Agents 59:106480. doi:10.1016/j.ijantimicag.2021.106480 34801675

[5] Odoi JO, Takayanagi S, Sugiyama M, Usui M, Tamura Y, Asai T. 2021. Prevalence of colistin-resistant bacteria among retail meats in Japan. Food Saf (Tokyo) 9:48–56. doi:10.14252/foodsafetyfscj.D-21-00002 34249589 PMC8254848

[6] Kawahara R, Fujiya Y, Yamaguchi T, Khong DT, Nguyen TN, Tran HT, Yamamoto Y. 2019. Most domestic livestock possess colistin-resistant commensal Escherichia coli harboring mcr in a rural community in Vietnam. Antimicrob Agents Chemother 63:e00594-19. doi:10.1128/AAC.00594-19 30988145 PMC6535556

[7] Liakopoulos A, Mevius DJ, Olsen B, Bonnedahl J. 2016. The colistin resistance mcr-1 gene is going wild. J Antimicrob Chemother 71:2335–2336. doi:10.1093/jac/dkw262 27330067

[8] Ruzauskas M, Vaskeviciute L. 2016. Detection of the mcr-1 gene in Escherichia coli prevalent in the migratory bird species Larus argentatus. J Antimicrob Chemother 71:2333–2334. doi:10.1093/jac/dkw245 27330066

[9] Ahlstrom CA, Ramey AM, Woksepp H, Bonnedahl J. 2019. Early emergence of mcr-1-positive Enterobacteriaceae in gulls from Spain and Portugal. Environ Microbiol Rep 11:669–671. doi:10.1111/1758-2229.12779 31216374

[10] Franklin AB, Ramey AM, Bentler KT, Barrett NL, McCurdy LM, Ahlstrom CA, Bonnedahl J, Shriner SA, Chandler JC. 2020. Gulls as sources of environmental contamination by colistin-resistant bacteria. Sci Rep 10:4408. doi:10.1038/s41598-020-61318-2 32157139 PMC7064522

[11] BirdLife International. 2024. Species factsheet: Larus schistisagus. https://datazone.birdlife.org/species/factsheet/slaty-backed-gull-larus-schistisagus.

[12] Yuan Y, Liang B, Jiang B-W, Zhu L-W, Wang T-C, Li Y-G, Liu J, Guo X-J, Ji X, Sun Y. 2021. Migratory wild birds carrying multidrug-resistant Escherichia coli as potential transmitters of antimicrobial resistance in China. PLoS ONE 16:e0261444. doi:10.1371/journal.pone.0261444 34910771 PMC8673662

[13] Rebelo AR, Bortolaia V, Kjeldgaard JS, Pedersen SK, Leekitcharoenphon P, Hansen IM, Guerra B, Malorny B, Borowiak M, Hammerl JA, Battisti A, Franco A, Alba P, Perrin-Guyomard A, Granier SA, De Frutos Escobar C, Malhotra-Kumar S, Villa L, Carattoli A, Hendriksen RS. 2018. Multiplex PCR for detection of plasmid-mediated colistin resistance determinants, mcr-1, mcr-2, mcr-3, mcr-4 and mcr-5 for surveillance purposes. Euro Surveill 23:17-00672. doi:10.2807/1560-7917.ES.2018.23.6.17-00672 PMC5824125 29439754

[14] Borowiak M, Baumann B, Fischer J, Thomas K, Deneke C, Hammerl JA, Szabo I, Malorny B. 2020. Development of a novel mcr-6 to mcr-9 multiplex PCR and assessment of mcr-1 to mcr-9 occurrence in colistin-resistant Salmonella enterica isolates from environment, feed, animals and food (2011–2018) in Germany. Front Microbiol 11:80. doi:10.3389/fmicb.2020.00080 32117115 PMC7011100

[15] Li J, Shi X, Yin W, Wang Y, Shen Z, Ding S, Wang S. 2017. A multiplex SYBR green real-time PCR assay for the detection of three colistin resistance genes from cultured bacteria, feces, and environment samples. Front Microbiol 8:2078. doi:10.3389/fmicb.2017.02078 29163387 PMC5663727

[16] Hamel M, Rolain JM, Baron SA. 2021. The history of colistin resistance mechanisms in bacteria: progress and challenges. Microorganisms 9:442. doi:10.3390/microorganisms9020442 33672663 PMC7924381

[17] Sato Y, Akasaka T, Yabuhara Y, Kazama K, Kawaguchi Y. 2020. Sensitivity mapping for seabirds and offshore wind power farms: a case study of Slaty-backed gulls in Hokkaido. J Conserv Ecol 25:191–203. doi:10.18960/hozen.1928

