# Supplementary figures and images for: Colistin-resistant Escherichia coli harboring mcr-1 in slaty-backed gull breeding in Northern Japan
Source: Microbiol Spectr. 2024 Oct 31;12(12):e00703-24. doi: 10.1128/spectrum.00703-24 (PMC11619456; doi:10.1128/spectrum.00703-24)

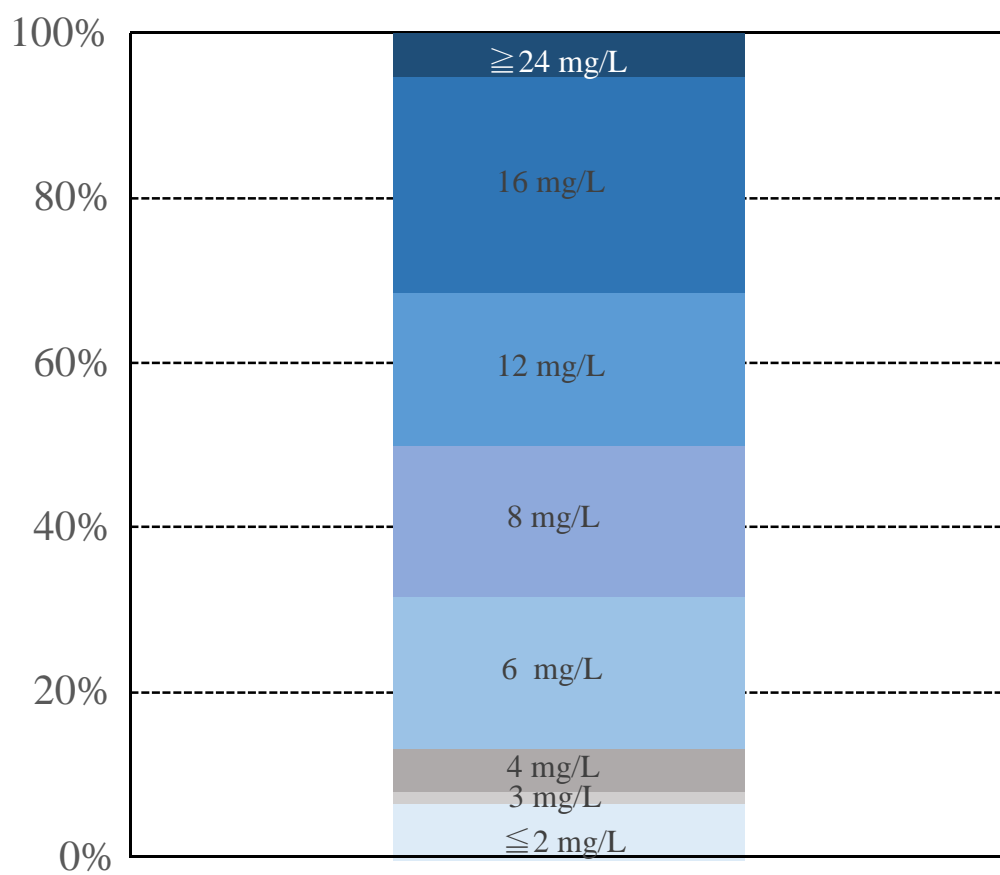

Figure S1. Distribution of colistin MICs in isolates from Slaty-backed Gulls.

Supplement: Figure S1 — Distribution of colistin MICs in isolates from Slaty-backed Gulls. [file spectrum.00703-24-s0001.pdf]
